# *GenomeFingerprinter*: The Genome Fingerprint and the Universal Genome Fingerprint Analysis for Systematic Comparative Genomics

**DOI:** 10.1371/journal.pone.0077912

**Published:** 2013-10-29

**Authors:** Yuncan Ai, Hannan Ai, Fanmei Meng, Lei Zhao

**Affiliations:** 1 State Key Laboratory for Biocontrol, School of Life Sciences, Sun Yat-sen University, Guangzhou, P. R. China; 2 Allergy Research Branch, State Key Laboratory of Respiratory Disease, The Second Affiliated Hospital, Guangzhou Medical University, Guangzhou, P. R. China; Dana-Farber Cancer Institute, United States of America

## Abstract

**Background:**

No attention has been paid on comparing a set of genome sequences crossing genetic components and biological categories with far divergence over large size range. We define it as the systematic comparative genomics and aim to develop the methodology.

**Results:**

First, we create a method, *GenomeFingerprinter*, to unambiguously produce a set of three-dimensional coordinates from a sequence, followed by one three-dimensional plot and six two-dimensional trajectory projections, to illustrate the genome fingerprint of a given genome sequence. Second, we develop a set of concepts and tools, and thereby establish a method called the universal genome fingerprint analysis (UGFA). Particularly, we define the total genetic component configuration (TGCC) (including chromosome, plasmid, and phage) for describing a strain as a systematic unit, the universal genome fingerprint map (UGFM) of TGCC for differentiating strains as a universal system, and the systematic comparative genomics (SCG) for comparing a set of genomes crossing genetic components and biological categories. Third, we construct a method of quantitative analysis to compare two genomes by using the outcome dataset of genome fingerprint analysis. Specifically, we define the geometric center and its geometric mean for a given genome fingerprint map, followed by the Euclidean distance, the differentiate rate, and the weighted differentiate rate to quantitatively describe the difference between two genomes of comparison. Moreover, we demonstrate the applications through case studies on various genome sequences, giving tremendous insights into the critical issues in microbial genomics and taxonomy.

**Conclusions:**

We have created a method, *GenomeFingerprinter*, for rapidly computing, geometrically visualizing, intuitively comparing a set of genomes at genome fingerprint level, and hence established a method called the universal genome fingerprint analysis, as well as developed a method of quantitative analysis of the outcome dataset. These have set up the methodology of systematic comparative genomics based on the genome fingerprint analysis.

## Introduction

By using conventional methods based on pair-wisely base-to-base comparison, comparing whole-genome sequences at large scale has not been achieved; even no attention was paid on handling a number of genomes crossing genetic components (chromosomes, plasmids, and phages) and biological categories (bacteria, archaeal bacteria, and viruses) with far divergence over large size range. We define such comparisons as the systematic comparative genomics. We believe it should be a priority task to carry out whole-genome-wide comparative genomics at large scale based on the geometrical analysis of sequences crossing diverse genetic components and biological categories in the post-genomic era. However, even simply visualizing a DNA sequence has been challenging for decades; little progress has been made to date [1].

Pioneering works in geometrical visualizing DNA sequences using computers had been done in one-dimension [2], [3], two-dimensions (Z-curve) [4], and three-dimensions (H-curve) [5], [6]. However, those were valid only for ‘static’ modeling and visualizing. The ‘dynamic’ modeling and visualizing had been explored in a virtual reality environment [7], [8]. AND-viewer, for example, provided a three-dimensional sensing of a big picture of a DNA sequence in a virtual reality environment by using a hand-sensor instead of mouse and keyboard [7], [8]. This pioneering work made fantastic progress in dynamically mimicking 3D visions and intuitively sensing genome sequences [7], [8]. Still, there was no possibility of using the outcome dataset to further explore the real contexts of biology.

The post-genomic era promoted a huge demand for data mining and robust reasoning with massive genome sequences [1]. So far, there were numerous methods for comparative genomics at small scale. These methods were divided into two types: algebraic approach [9], [10], [11], [12] and geometrical approach [13].

The algebraic approach means that the calculation of similarity or identity is based on pair-wisely base-to-base comparison. The output dataset is only used for visualization through graphical techniques [1]. The most common tools were BLAST [9] and CLUSTALW [10]. Recently, a BLAST-based tool, BRIG, was constructed for genome-wide comparison to create images of multiple circular genomes among a number of very closely related bacteria strains [11]. The output image showed the BLAST-similarity between one central reference sequence and other inquiry sequences as a set of concentric rings, in which BLAST-matches were colored on a sliding scale indicating a defined percentage of BLAST-identity. This tool had great advantages over other common tools, like ACT [12], in terms of the numbers of genomes being simultaneously compared and the ways of presenting its output images. These features made it a versatile tool for visualizing a range of genome data, but it was still only for visualization. Similarly, the Mauve program [14], [15], combining both algebraic calculation and graphic display, was widely used for comparing and visualizing a set of genomes. However, even within close relatives, the number of genomes being handled by Mauve was dramatically dependent on the computational constraints, taking up too much CPU time or causing memory overflow, which limited Mauve to handle few very close relatives at one time.

The geometrical approach means that a genome sequence can be transformed into a set of coordinates to be plotted giving a geometrical vision. Most importantly, both calculation and visualization are separately processed in a dynamic way so that the input and output can be subsequently re-useable for geometrical analysis. One promising example was the Z-curve method (Zplotter program), which generated a set of three-dimensional coordinates from a linear genome sequence [16]. Such coordinates were plotted to create three-dimensional geometrical visions (as open rough Z-curves) for the given DNA sequences [16]. Hundreds of such visions for microbial genomes were collected as a database [17]. The Z-curve method (Zplotter program) was used not only for visualization but for geometrical analysis to explore the real contexts of biology [18], [19], [20], [21]. For example, two replication *ori* points in archaeal bacterial genomes were predicted by the Z-curve analysis [22], [23] and confirmed by the wet experiments in other labs [24], [25], thus showing it’s promising. However, the Zplotter algorithm had an inevitable flaw to falsely present a genome sequence due to its ambiguous cutting-point error (see Discussion section), which was not be suitable for creating a stable unique genome fingerprint, as we proposed; nonetheless, no statistic analysis could be further applied to the outcome dataset.

In this paper, we present a method called *GenomeFingerprinter* to unambiguously produce a unique set of three-dimensional coordinates from a sequence, followed by one three-dimensional plot and six two-dimensional trajectory projections, to illustrate the whole-genome fingerprint of a given genome sequence. We further develop a set of concepts and tools, and thereby establish a method called the universal genome fingerprint analysis (UGFA). Finally, we construct a method to quantitatively analyze the outcome dataset of genome fingerprint analysis. Moreover, we demonstrate the applications of such methods through various case studies, giving new insights into the critical issues in microbial genomics and taxonomy. These have set up the methodology of what we called the systematic comparative genomics based on the genome fingerprint and the universal genome fingerprint analysis. We anticipate that these comprehensive methods can be widely applied at large scale in the post-genomic era.

## Results

### 1. Mathematical Model and Three-dimensional Coordinate

To geometrically visualize a sequence, the key step is to create a set of three-dimensional coordinates (x_n_, y_n_, z_n_) for each base. To do this, the Z-curve method (Zplotter program) [16] defined a set of coordinates (x_n_, y_n_, z_n_) for each base in a linear sequence (n = 1, 2, …, N; N is the sequence length) by the equation (**0**), which defined a unique Z-curve for a given linear sequence and *vice versa*. Note that A_n_, T_n_, G_n_, C_n_ were the sum of total numbers of each of four base-type (A, T, G, C), respectively, counting from the first base to the bases before the first base (passing through the n^th^ base in the process) in a linear sequence (n = 1, 2, …, N). However, the main problem was the ambiguity of the “first base” due to cutting-point error in a deposited sequence (see explanations in Discussion section).
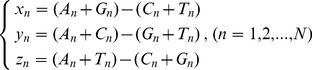
(0)


**Figure 1:**
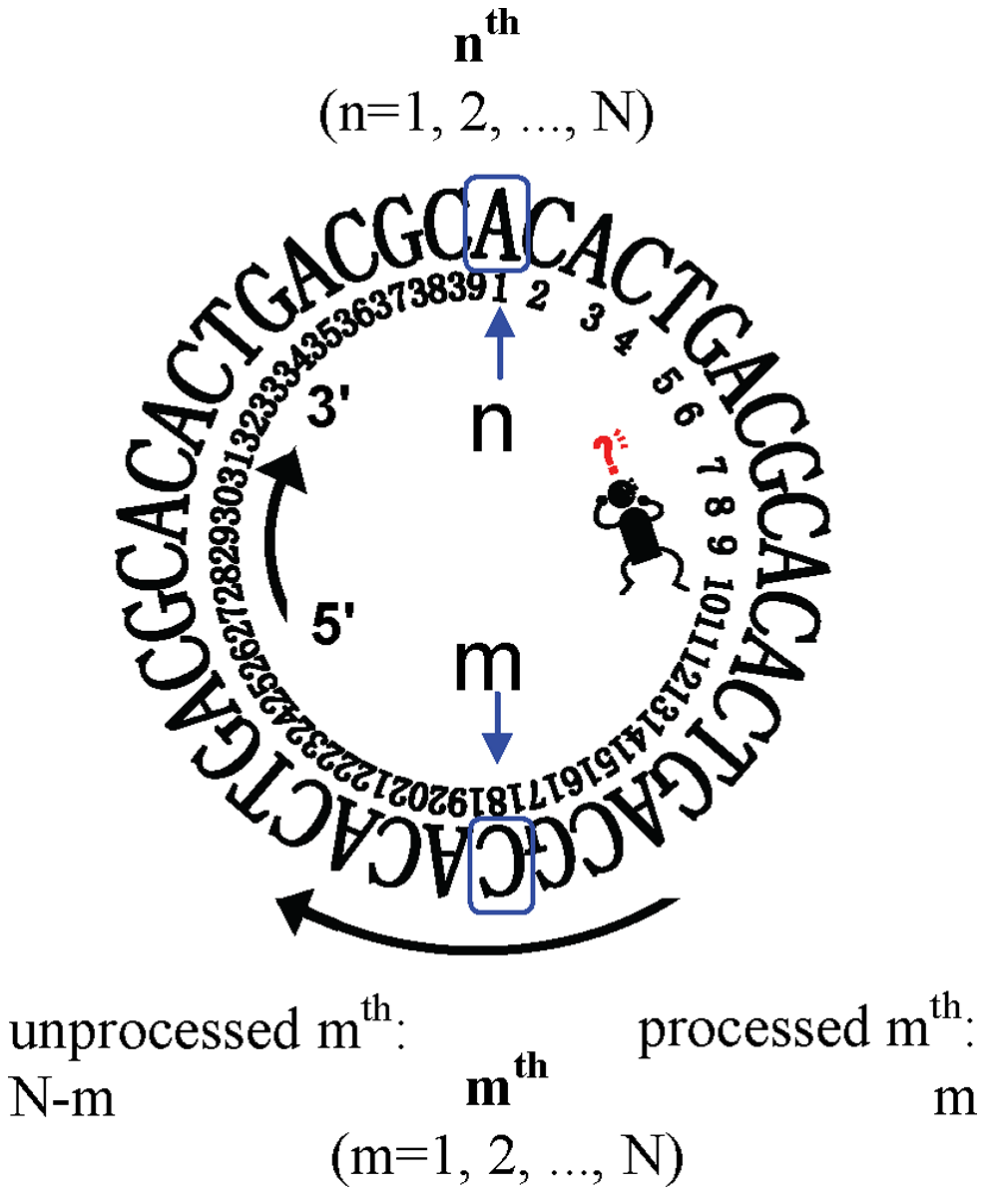
A mathematical model for creating a set of coordinates (x_n_, y_n_, z_n_) from a circular genome sequence. We randomly select a base (the n^th^) as the first target base (TB) while keep moving the m^th^ focusing base (FB). For the given TB (n^th^), we define the relative distance (RD) between the selected TB (n^th^) and the moving FB (m^th^) (m = 1, 2, …, N).

Here we take the same defining form as the equation (**0**), but with different contents of A_n_, T_n_, G_n_, C_n_. Namely, we propose a model called *GenomeFingerprinter* for the geometrical visualization of a circular sequence. As an artificial example, a circular sequence containing 40-bps, (5′-3′) ACACTGACGCACACTGACGCACACTGACGCACACTGACGC (Figure 1), will be used to illustrate the conceptual framework. It will be described in reasonable detail in order to build a bridge for the readers who may not have multiple disciplinary backgrounds [26].

First, we randomly select a base (the n^th^) as the first targeted base (TB) while keep the m^th^ focusing base (FB) moving. We define the relative distance (RD) (**1**) between the selected TB (n^th^) and the moving FB (m^th^) (m = 1, 2, …, N).
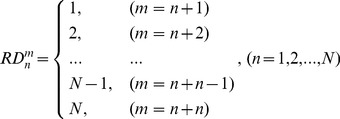
(1)


Note that the RD concept is extremely critical. The RD formula (**1**) can virtually treat an arbitrary linear sequence as a circular one. For example, once we select the TB (e.g., suppose at position 1, base **A**) and the moving FB (e.g., suppose at position 20, base **C**), the RD value is 19 (Figure 1). Thus, a collection of RD values (m = 1, 2, …, N) can be generated for each selected TB (in total N number) sliding along with the given sequence. Particularly, the RD value is N, not zero, when the m^th^ FB is located at the same position with TB, which means the m^th^ FB has gone through one circle (i.e., starting from and finishing at the same position at the n^th^ base).

Second, we define the weighted relative distance (WRD) (**2**). The above value (base **C** at position 20), for example, is 19/40. This is simply for reducing memory burden and overcoming computational constraints for large sequences.
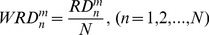
(2)


Third, for the same selected TB (n^th^), we define the sum of the weighted relative distance (SWRD) (**3**) from the collection of WRD (m = 1, 2, …, N) for each of four base-type (A, G, T, C), respectively.
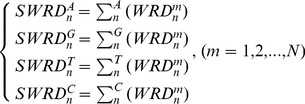
(3)


Fourth, we define a set of coordinates (x_n_, y_n_, z_n_) (**4**) for the selected TB (n^th^). Note that we count the sum of the weighted relative distance (SWRD) (unlike the Zplotter program counting the sum of numbers) for each of four base-type (A, T, G, C), respectively. So far, only one cycle has been done for only one selected TB (n^th^); namely, only one base has had its coordinates (x_n_, y_n_, z_n_).
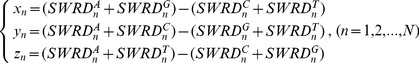
(4)


Finally, we repeat the above steps to create a set of coordinates for every base in the sequence. Briefly, by selecting the next TB (e.g., n = 2) and reiterating the processes for each base, step-by-step, we will finish the N cycles (n = 1, 2, …, N); and each cycle has one selected TB, which will create one set of coordinates (x_n_, y_n_, z_n_) for that chosen TB. Ultimately, after having finished the total N cycles, all N bases of the sequence will have their own coordinates so that a series of sets of coordinates (x_n_, y_n_, z_n_) will be created for the genome sequence. We have developed an in-house script, GenomeFingerprinter.exe to do all. Note that our method is also valid for RNA by simply replacing T with U base.

As an example, by using our program GenomeFingerprinter.exe, we can calculate a series of coordinates (x_n_, y_n_, z_n_) for the artificial genome sequence containing 40-bps (Figure 1); there are total 40 bases and each base has its own coordinates (x_n_, y_n_, z_n_) (data not shown).

### 2. Three-dimensional Plot and the Primary Genome Fingerprint Map

**Figure 2:**
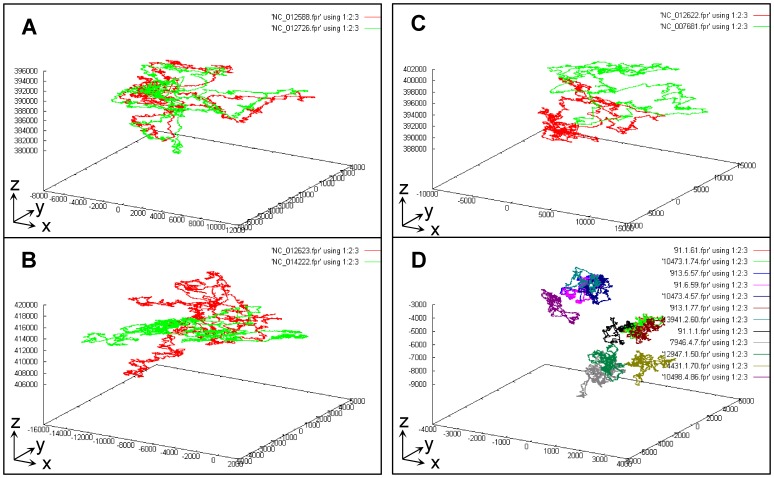
The primary genome fingerprint map (P-GFM) for the overall comparison among a number of genome fingerprint maps. (A). Similar: *Sulfolobus islandicus* M.14.25 (NC_012588) and M.16.4 (NC_012726); (B). Partly similar: *S. islandicus* Y.N.15.51 (NC_012623) and *Methanococcus voltae* A3 (NC_014222); (C). Different: *S. islandicus* Y.G.57.14 (NC_012622) and *Methanosphaera stadtmanae* 3091 (NC_007681); (D). Mixture: (twelve fragmental genomes of strains in *Escherichia coli* (listed in Table 1): 91.1.1, 91.1.61, 91.6.59, 913.1.77, 913.5.57, 4431.1.70, 7946.4.7, 10473.1.74, 10473.4.57, 10498.4.86, 12947.1.50, 13941.2.60.

The set of coordinates (x_n_, y_n_, z_n_) of a given sequence can be plotted as a three-dimensional plot (3D-P) to give a geometrical vision. As an example, the artificial sequence (Figure 1) has only 40 points giving a naive vision (not shown). Instead, we show the real visions of strains from bacteria and archaeal bacteria (Table 1) (Figure 2). Clearly, each vision (Figure 2) has its individual genome fingerprint (GF). We define such a GF vision as the genome fingerprint map (GFM), which is an intuitive identity or a unique digital marker for a given genome sequence. For convenience, we further define such a GFM vision of three-dimensional plot as the primary genome fingerprint map (P-GFM). Therefore, from now on, we can directly operate and compare the GFM vision for comparing sequences. In other words, we compare genome sequences through the genome fingerprints (via geometrical analysis) instead of the sequence base-pairs (via algebraic analysis).

**Table 1:** Features of genome sequences from bacteria and archaeal bacteria.

Species and Strain	Sequence ID	Type	Size (bps)
**Downloaded from FTP.ncbi.nlm.nih.gov [GenBank]**			
*Escherichia coli* K-12/W3110	AC_000091NC_007779	Chromosome	4646332
*Escherichia coli* K-12/DH10B	NC_010473	Chromosome	4686137
*Escherichia coli* K-12/MG1655	NC_000913	Chromosome	4639675
*Escherichia coli* BL21 (DE3) pLysSAG	NC_012947	Chromosome	4570938
*Escherichia coli* O55:H7/CB9615	NC_013941	Chromosome	5386352
*Escherichia coli* UTI89	NC_007946	Chromosome	5065741
*Escherichia coli* CFT073	NC_004431	Chromosome	5231428
*Escherichia coli* SMS-3-5	NC_010498	Chromosome	5068389
*Sulfolobus islandicus* M.14.25	NC_012588	Chromosome	2608832
*Sulfolobus islandicus* M.16.4	NC_012726	Chromosome	2586647
*Sulfolobus islandicus* Y.N.15.51	NC_012623	Chromosome	2812165
*Sulfolobus islandicus* Y.G.57.14	NC_012622	Chromosome	2702058
*Methanococcus voltae* A3	NC_014222	Chromosome	1936387
*Methanosphaera stadtmanae* DSM 3091	NC_007681	Chromosome	1767403
*Halomonas elongate* DSM 2581	NC_014532	Chromosome	4119315
*Halorhodospira halophilia* SL1	NC_008789	Chromosome	2716716
*Halorhabdus utahensis* DSM 12940	NC_013158	Chromosome	3161321
*Halothermothrix orenii* H 168	NC_011899	Chromosome	2614977
*Halothiobacillus neapolitanus* c2	NC_013422	Chromosome	2619785
*Halogeometricum boringquense* DSM 11551	NC_014729	Chromosome	2860838
*Haloterrigena turkmenica* DSM 5511	NC_013743	Chromosome	3944596
*Natrinema pellirubrum* DSM 15624	NC_019962	Chromosome	3844629
*Haloquadratum walsbyi* DSM 16790	NC_008212	Chromosome	3177244
*Halorubrum lacusprofundii* ATCC49239	NC_012029	Chromosome	2774371
*Halorubrum lacusprofundii* ATCC49239	NC_012028	Chromosome	533457
*Haloarcula marismortui* ATCC43049	NC_006396	Chromosome	3176463
*Haloarcula marismortui* ATCC43049	NC_006397	Chromosome	292165
*Haloarcula marismortui* ATCC43049	NC_006389	plasmid pNG100	33779
*Haloarcula marismortui* ATCC43049	NC_006390	plasmid pNG200	33930
*Haloarcula marismortui* ATCC43049	NC_006391	plasmid pNG300	40086
*Haloarcula marismortui* ATCC43049	NC_006392	plasmid pNG400	50776
*Haloarcula marismortui* ATCC43049	NC_006393	plasmid pNG500	134574
*Haloarcula marismortui* ATCC43049	NC_006394	plasmid pNG600	157519
*Haloarcula marismortui* ATCC43049	NC_006395	plasmid pNG700	416420
*Halomicrobium mukohataei* DSM 12286	NC_013202	Chromosome	3154923
*Halomicrobium mukohataei* DSM 12286	NC_013201	plasmid pHmuk01	225032
*Haloferax vocanii* DS2	NC_013967	Chromosome	2888440
*Haloferax vocanii* DS2	NC_013964	plasmid pHV3	444162
*Haloferax vocanii* DS2	NC_013965	plasmid pHV2	6450
*Haloferax vocanii* DS2	NC_013966	plasmid pHV4	644869
*Haloferax vocanii* DS2	NC_013968	plasmid pHV1	86308
*Halobacterium* sp.NRC-1	NC_002607	Chromosome	2014239
*Halobacterium salinarum* R1	NC_010364	Chromosome	2000962
**Derivatives created in this study [based on those sequences from GenBank]**			
*Escherichia coli* K-12/W3110-91.1.1	91.1.1	Chromosome fragment	227694
*Escherichia coli* K-12/W3110-91.1.61	91.1.61	Chromosome fragment	324260
*Escherichia coli* K-12/W3110-91.6.59	91.6.59	Chromosome fragment	410186
*Escherichia coli* K-12/W3110-91.F7	91.7	Chromosome fragment	953958
*Escherichia coli* K-12/MG1655-913.1.77	913.1.77	Chromosome fragment	331163
*Escherichia coli* K-12/MG1655-913.5.57	913.5.57	Chromosome fragment	408963
*Escherichia coli* CFT073-4431.1.70	4431.1.70	Chromosome fragment	401260
*Escherichia coli* UTI89-7946.4.7	7946.4.7	Chromosome fragment	518065
*Escherichia coli* K-12/DH10B -10473.1.74	10473.1.74	Chromosome fragment	325622
*Escherichia coli* K-12/DH10B -10473.4.57	10473.4.57	Chromosome fragment	412818
*Escherichia coli* SMS-3-5-10498.4.86	10498.4.86	Chromosome fragment	331536
*Escherichia coli* BL21 (DE3) pLysSAG-12947.F1	12947.1	Chromosome fragment	1759795
*Escherichia coli* BL21 (DE3) pLysSAG-12947.1.50	12947.1.50	Chromosome fragment	470050
*Escherichia coli* BL21 (DE3) pLysSAG-12947.F5	12947.5	Chromosome fragment	43254
*Escherichia coli* O55:H7/CB9615-13941.F1	13941.1	Chromosome fragment	1915479
*Escherichia coli* O55:H7/CB9615-13941.2.60	13941.2.60	Chromosome fragment	267039

For instance, we can intuitively distinguish a number of genome sequences based on their genome fingerprint maps (Figure 2). Within the same species *Sulfolobus islandicus*, strains M.14.25 and M.16.4 share similarity (Figure 2, A), indicating subtle variations at strain level. With far divergence, however, strain *S. islandicus* Y.N.15.51 differs globally from *Methanococcus voltae* A3 but shares local similar regions (Figure 2, B); whereas *S. islandicus* Y.G.57.14 completely differs from *Methanosphaera stadtmanae* DSM 3091 (Figure 2, C), confirming their farther divergences beyond genus level.

### 3. Two-dimensional Trajectory Projections and the Secondary Genome Fingerprint Maps

**Figure 3:**
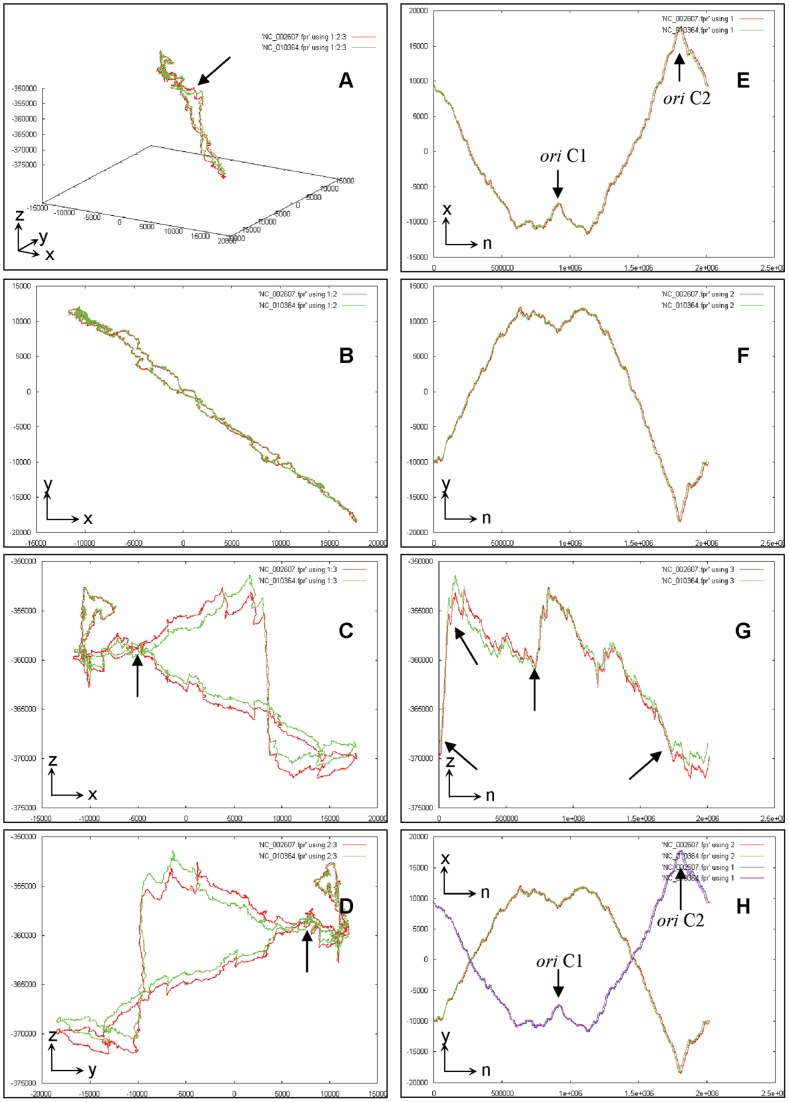
The primary genome fingerprint map (P-GFM) (A) and the secondary genome fingerprint maps (S-GFMs) (B∼H) for the comparisons between two chromosomes of *Halobacterium* sp. NRC-1 (NC_002607) and *Halobacterium salinarum* R1 (NC_010364). (A). x_n_∼y_n_∼z_n_; (B). x_n_∼y_n_; (C). x_n_∼z_n_; (D). y_n_∼z_n_; (E). x_n_∼n; (F). y_n_∼n; (G). z_n_∼n; (H). x_n_∼n and y_n_∼n together. Note that two replication *ori* points (*ori*C1 and *ori* C2) are marked by arrows; other arrows indicated the genome-wide evolution events.

To demonstrate the genome fingerprint in a more sophisticated way, we further create six two-dimensional trajectory projections (2D-TPs) for a given P-GFM through six combinations (x_n_∼n, y_n_∼n, z_n_∼n, x_n_∼y_n_, x_n_∼z_n_, and y_n_∼z_n_) of the coordinates. For convenience, such six 2D-TPs are defined as the secondary genome fingerprint maps (S-GFMs). For example, the six S-GFMs comparing two chromosomes between *Halobacterium* sp. NRC-1 (NC_002607) and *Halobacterium salinarum* R1 (NC_010364) clearly demonstrate the subtle variations both globally and locally (Figure 3). Note that the S-GFMs of x_n_∼z_n_, y_n_∼z_n_, x_n_∼y_n_ usually carry much more sensitive information than those of x_n_∼n, y_n_∼n, and z_n_∼n do, respectively. Accordingly, the S-GFMs can amplify subtle variations that usually are insensitive or invisible in the P-GFMs. In particular, the S-GFMs of x_n_∼y_n_, x_n_∼z_n_ and y_n_∼z_n_ are much more sensitive in differentiating the local subtle variations and identifying the unique genome features; whereas the S-GFMs of x_n_∼n, y_n_∼n and z_n_∼n are relatively less informative but still useful when focusing on global patterns (Figure 3).

### 4. The Universal Genome Fingerprint Map (UGFM)

**Figure 4:**
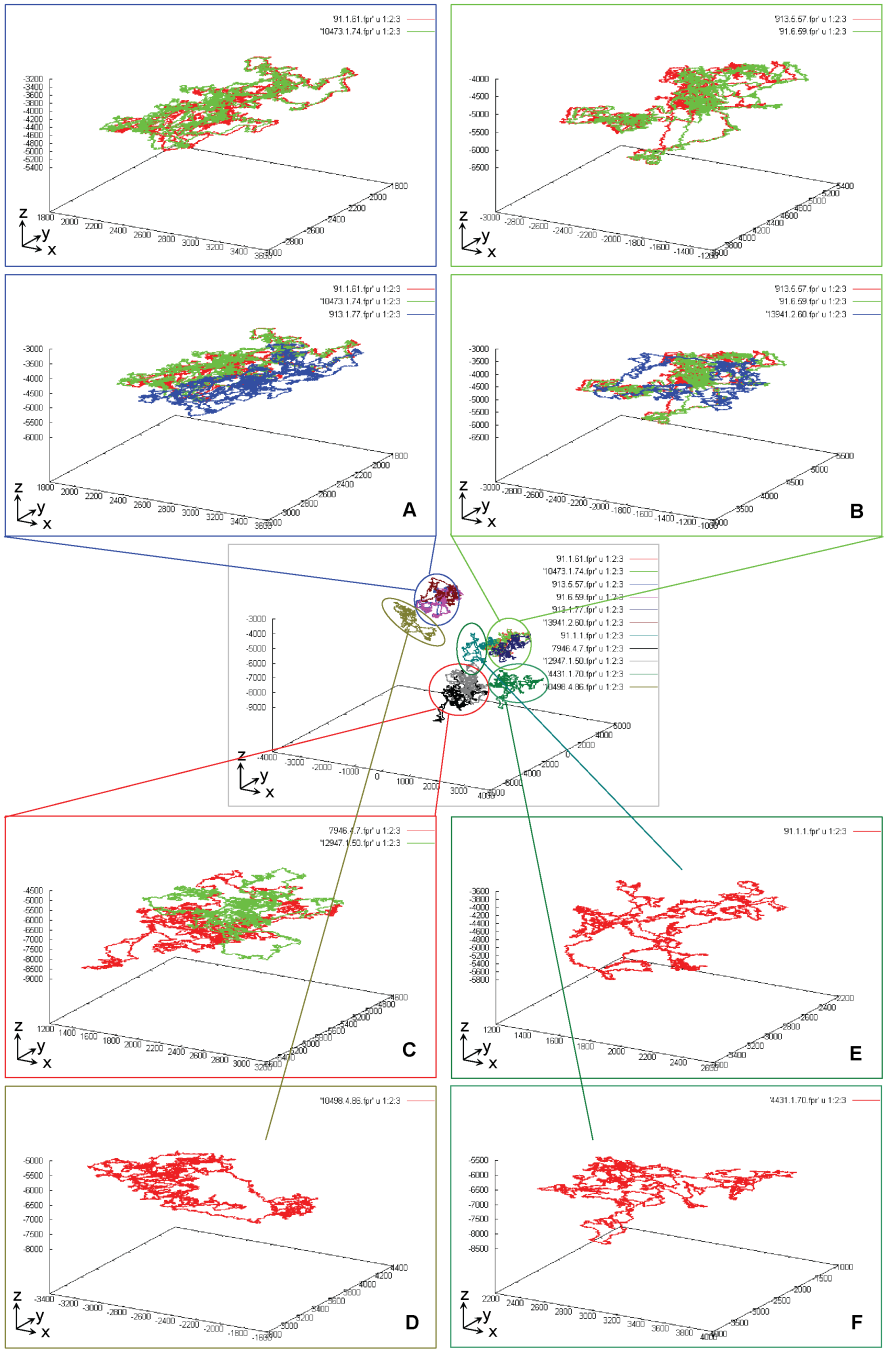
The universal genome fingerprint map (UGFM) for the comparison among a set of genomes in-one-sitting. Twelve fragmental genome sequences (Table 1) are shown as one UGFM vision. Each individual primary genome fingerprint map (P-GFM) is classified into a discrete group solely based on its location: Group (A) (91.1.61, 913.1.77 and 10473.1.74), Group (B) (91.6.59, 913.5.57 and 13941.2.60), Group (C) (7946.4.7 and 12947.1.50), Group (D) (10498.4.86), Group (E) (91.1.1), and Group (F) (4431.1.70).

As shown in Figure 3, for convenience, we further define the universal genome fingerprint map (UGFM) to unify both P-GFM and S-GFMs for the comparison in-one-sitting. Namely, we can compare a number of sequences through displaying their multiple GFMs (regardless of P-GFMs or S-GFMs) at one time (in-one-sitting) as one UGFM vision; from that, each individual GFM can be classified into a discrete group solely based on its location. For example, those P-GFMs (Figure 2, D) of the twelve fragmental genomes from eight strains of *E.coli* (Table 1) are enlarged and displayed on one UGFM vision, and classified into six discrete groups (Figure 4).

Clearly, there are six groups on the UGFM vision (Figure 4, A, B, C, D, E, F). Particularly, different fragmental genome sequences either from the same strain (e.g., 91.1.1, 91.1.61, 91.6.59) or from different strains (e.g., 913.5.57, 4431.1.70, 7946.4.7, 10473.1.74, 10498.4.86, 12947.1.50, 13941.2.60) (Table 1) can be revealed by the complex P-GFM patterns. Some are similar including (91.1.61, 913.1.77,10473.1.74) (Figure 4, A) and (91.6.59, 913.5.57,13941.2.60) (Figure 4, B), but most are different (Figure 4, C, D, E, F). These data likely indicate the existence of modular domains in genomes; and such mosaic structures likely reveal their evolutionary history.

Moreover, note that a given P-GFM vision has quite different views between its own format and that of the UGFM vision (Figure 4), simply because of what we called the effects of scale-down and view-angle rotation in the UGFM vision. This feature could ensure the UGFM vision to be a powerful tool for global comparison at large scale. Namely, as many sequences as possible could be handled at one time (in-one-sitting) as long as the computer memory and the graphic software could allot.

### 5. The Universal Genome Fingerprint Analysis (UGFA)

**Figure 5:**
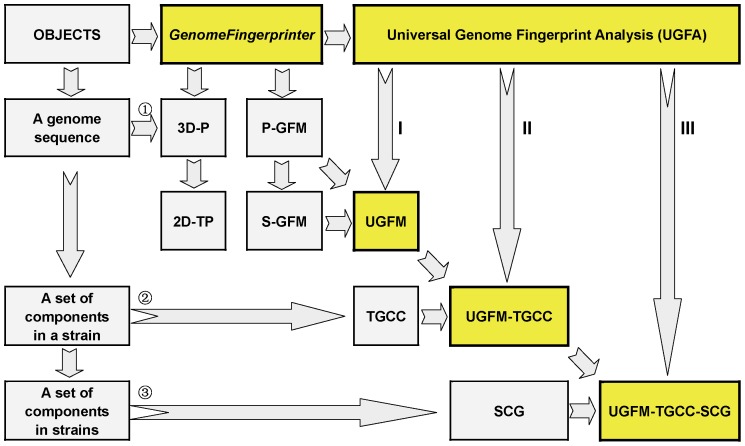
The conceptual framework of the universal genome fingerprint analysis (UGFA). The core concepts and tools include UGFM, UGFM-TGCC, and UGFM-TGCC-SCG. Abbreviations: 3D-P: three-dimensional plot; 2D-TP: two-dimensional trajectory projections; GF: genome fingerprint; GFM: genome fingerprint map; P-GFM: primary genome fingerprint map; S-GFM: secondary genome fingerprint map; UGFM: universal genome fingerprint map; TGCC: total genetic component configuration; UGFM-TGCC: universal genome fingerprint map of total genetic component configuration; SCG: systematic comparative genomics; UGFM-TGCC-SCG: universal genome fingerprint map of total genetic component configuration based systematic comparative genomics; UGFA: universal genome fingerprint analysis.

We further establish a method called the universal genome fingerprint analysis (UGFA) (Figure 5). Briefly, the UGFA method consists of a set of concepts and tools under three subcategories corresponding to three objects: a genome, a strain, and a set of strains, respectively. In other words, the objects of comparison can be one genome sequence, a number of genome sequences crossing genetic components (chromosomes, plasmids, and phages, if applicable) in a strain, or a set of genome sequences of genetic components in strains crossing biological categories (bacteria, archaeal bacteria, viruses). We anticipate that it should be effective for what we called the systematic comparative genomics at large scale, by expanding the scope of genetic component and biological category as well as the power of computation.

#### 5.1. UGFM

First, the UGFM tool, namely the universal genome fingerprint map (UGFM), is the foundation of the UGFA method. As shown earlier (Figure 3, 4), the UGFM (combined the P-GFM and the S-GFMs) has been proved powerful in the comparison among a number of genomes crossing both archaeal and prokaryote bacteria genomes.

#### 5.2. UGFM-TGCC

**Figure 6:**
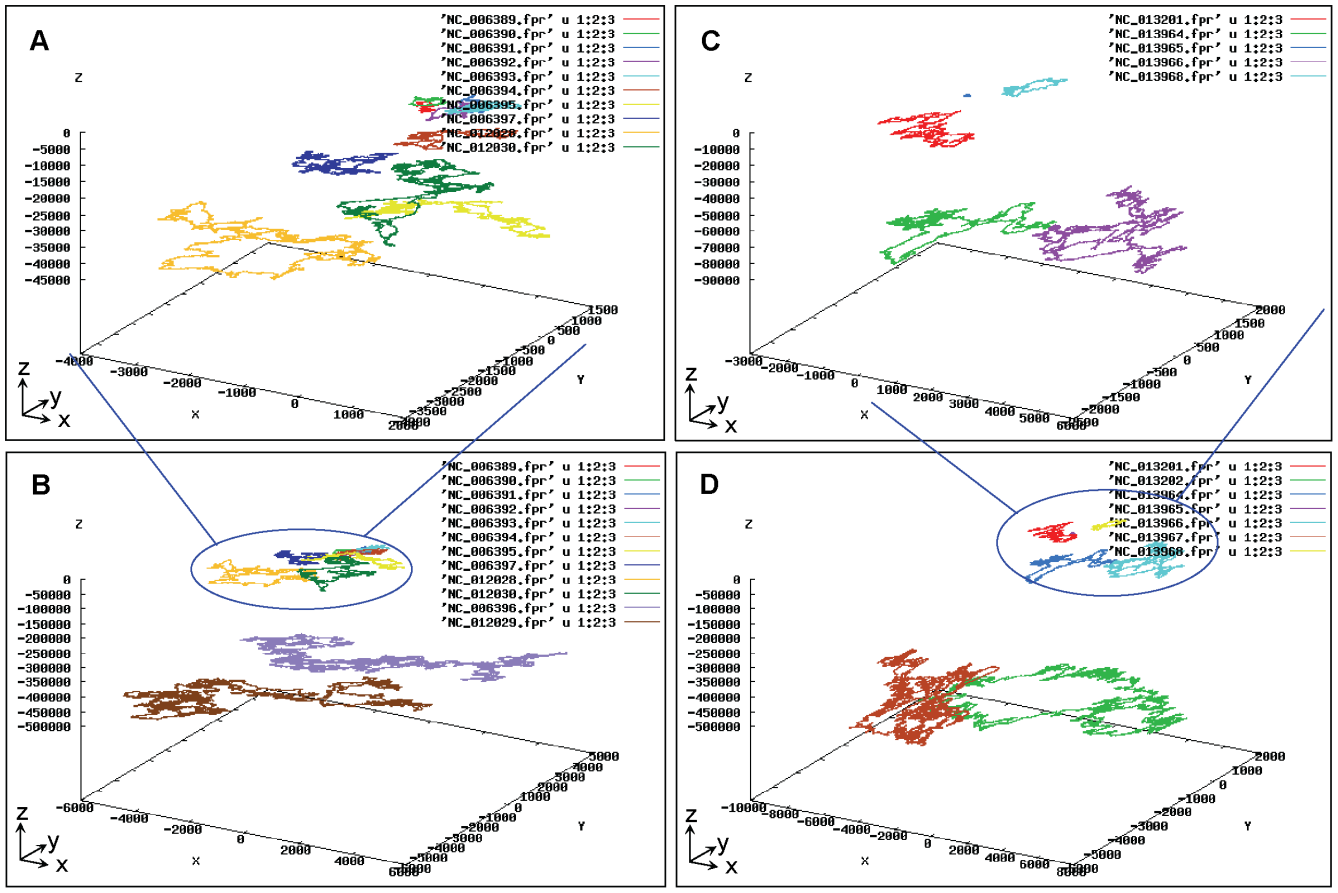
The UGFM-TGCC-SCG of four archaeal bacterial strains crossing four genera of halophilic Archaea. One set (A *vs*.B): *Halorubrum lacusprofundii* ATCC49239 [chromosome I (NC_012029), chromosome II (NC_012028), plasmid pHLAC01 (NC_012030)] *vs*. *Haloarcula marismortui* ATCC43049 [chromosome I (NC_006396), chromosome II (NC_006397), and seven plasmids pNG100 (NC_006389), pNG200 (NC_006390), pNG300 (NC_006391), pNG400 (NC_006392), pNG500 (NC_006393), pNG600 (NC_006394), pNG700 (NC_006395)] focusing on plasmids (A) and as a universal system (B); The other set (C *vs*.D): *Haloferax vocanii* DS2 [chromosome (NC_013967), and four plasmids pHV3 (NC_013964), pHV2 (NC_013965), pHV4 (NC_013966), pHV1 (NC_013968)] *vs*. *Halomicrobium mukohataei* DSM 12286 [chromosome (NC_013202), plasmid pHmuk01(NC_013201)] focusing on plasmids (C) and as a universal system (D). Note that the tiny spots and the giant visions are elegantly plotted in-one-sitting within the same figure.

Second, we define the total genetic component configuration (TGCC) for a set of genomes crossing genetic components (chromosomes, plasmids, and phages, if applicable) in a strain for describing the strain as a systematic unit. We further define the universal genome fingerprint map (UGFM) of the total genetic component configuration (TGCC) (UGFM-TGCC) for differentiating a set of genetic components in a strain as a universal system. Putting together, the UGFM-TGCC tool, namely the universal genome fingerprint map (UGFM) of the total genetic component configuration (TGCC), can be used to perform the comparison among a set of genomes crossing genetic components within a strain, which will be exemplified in the next section (Figure 6).

#### 5.3. UGFM-TGCC-SCG

Third, we define the UGFM-TGCC-SCG tool, namely UGFM-TGCC-based systematic comparative genomics (SCG), in order to compare a set of genomes crossing both genetic components (chromosomes, plasmids, and phages, if applicable) and biological categories (bacteria, archaeal bacteria, viruses) in a universal system.

At moderate scale, one example (Figure 6) demonstrates that nineteen genomes (including six chromosomes and thirteen plasmids) with large size range (6 Kbp∼4 Mbp) can be mapped and compared by using the UGFM-TGCC-SCG tool. These nineteen genomes from four strains (each containing at least one chromosome and one plasmid) crossing four genera of halophilic Archaea (Table 1) are compared as two sets (Figure 6): *Halorubrum lacusprofundii* ATCC 49239 (two chromosomes and one plasmid) *vs*. *Haloarcula marismortui* ATCC 43049 (two chromosomes and seven plasmids) (Figure 6, A, B); while *Haloferax vocanii* DS2 (one chromosome and four plasmids) *vs*. *Halomicrobium mukohataei* DSM 12286 (one chromosome and one plasmid) (Figure 6, C, D). Obviously, they are shown quite divergent solely based on their genome fingerprint maps on the UGFM-TGCC-SCG visions. Most importantly, the tiny spots (e.g., corresponding to 6 Kbp) and the giant visions (e.g., corresponding to 4 Mbp) are harmoniously co-existed in the same figure, either closely or distantly.

**Figure 7:**
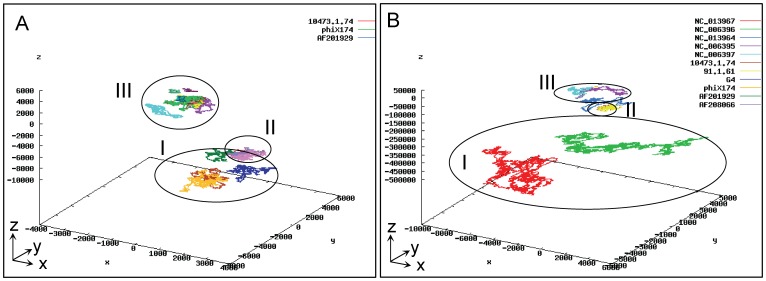
The landscape of the UGFM-TGCC-SCG visions at large scale. (A). The twelve bacterial fragmental chromosomes of *E.coli* (II) (Table 1), twenty four virus genomes (I) and forty seven phage genomes (III) (Table 2) are shown as three distinct groups, resulting in fewer maps because the genomes are very close relatives and accordingly almost repeat themselves; (B). The representatives selected from (A) are shown as three distinct groups: two archaeal bacterial chromosomes (I); two bacterial fragmental chromosomes of *E.coli*, two viruses, and two phages (II); three plasmids (III). The strong effects of scale-down and view-angle rotation at large scale are demonstrated.

At large scale, the UGFM-TGCC-SCG vision can demonstrate the amazing landscape of a large set of genomes both crossing diverse genetic components (chromosomes, plasmids, and phages) and crossing diverse biological categories (bacteria, archaeal bacteria, viruses). For instance, we make up a large set (over one hundred) of genomes of interest by combing 6 archaeal bacterial genomes and 13 archaeal bacterial plasmids (shown in Figure 6), 12 fragmental chromosomes of *E.coli* (shown in Figure 4), 47 phage genomes and 24 virus genomes (as listed in Table 2) to be compared at large scale by using the UGFM-TGCC-SCG tool. Remind that the effects of scale-down and view-angle rotation as demonstrated earlier (Figure 4) could ensure that as many sequences as possible could be handled at one time as long as the computer memory and the graphic software could allot. Under our conditions (physical 2-Gb memory and 32-bits graphic software), we can only handle up to 1.5 Gb data in-one-sitting. As such, we generate two sets, separately. One set contains eighty three genomes: 24 viruses (I), 12 fragmental chromosomes of *E.coli* (II), and 47 phages (III), which are shown as three distinct groups (Figure 7, A). The other set consists of two archaeal bacterial chromosomes (I), two bacterial fragmental chromosomes/two phages/two viruses (II), and three plasmids (III), which are shown as three distinct groups (Figure 7, B). These are generally consistent with their real biological distinctions at different taxonomical levels. Obviously, here the effects of scale-down and view-angle rotation are demonstrated even stronger than those in earlier sections. Moreover, in the big group of phages and viruses (II), most genomes seem as very close relatives and accordingly almost repeat themselves within the phage or virus subgroup, respectively, resulting in fewer maps than should be.

**Table 2:** Features of genome sequences from phages and viruses.

Species and Strain	Sequence ID	Type	Size (bps)
**Downloaded from FTP.ncbi.nlm.nih.gov [GenBank]**			
WA5: Coliphage WA5	NC_007847	Phage chromosome	5737
ID11: Coliphage ID11	NC_006954	Phage chromosome	5737
WA3: Coliphage WA3	NC_007845	Phage chromosome	5700
WA2: Coliphage WA2	NC_007844	Phage chromosome	5700
ID41: Coliphage ID41	NC_007851	Phage chromosome	5737
NC10: Coliphage NC10	NC_007854	Phage chromosome	5687
WA6: Coliphage WA6	NC_007852	Phage chromosome	5687
ID12: Coliphage ID12	NC_007853	Phage chromosome	5687
NC13: Coliphage NC13	NC_007849	Phage chromosome	5737
NC2: Coliphage NC2	NC_007848	Phage chromosome	5737
NC6: Coliphage NC6	NC_007855	Phage chromosome	5687
ID52: Coliphage ID52	NC_007825	Phage chromosome	5698
ID8: Coliphage ID8	NC_007846	Phage chromosome	5700
G4: Enterobacteria phage G4	NC_001420	Phage chromosome	5737
ID2: Coliphage ID2	NC_007817	Phage chromosome	5644
WA14: Coliphage WA14	NC_007857	Phage chromosome	5644
ID18: Coliphage ID18	NC_007856	Phage chromosome	5644
WA45: Coliphage WA45	NC_007822	Phage chromosome	6242
ID21: Coliphage ID21	NC_007818	Phage chromosome	6242
NC28: Coliphage NC28	NC_007823	Phage chromosome	6239
ID62: Coliphage ID62	NC_007824	Phage chromosome	6225
NC35: Coliphage NC35	NC_007820	Phage chromosome	6213
NC29: Coliphage NC29	NC_007827	Phage chromosome	6439
NC3: Coliphage NC3	NC_007826	Phage chromosome	6273
alpha3: Enterobacteria phage alpha3	DQ085810	Phage chromosome	6177
WA13: Coliphage WA13	NC_007821	Phage chromosome	6242
phiK: Coliphage phiK	NC_001730	Phage chromosome	6263
ID32: Coliphage ID32	NC_007819	Phage chromosome	6245
NC19: Coliphage NC19	NC_007850	Phage chromosome	5737
NC16: Coliphage NC16	NC_007836	Phage chromosome	5540
NC5: Coliphage NC5	NC_007833	Phage chromosome	5540
NC37: Coliphage NC37	NC_007837	Phage chromosome	5540
ID1: Coliphage ID1	NC_007828	Phage chromosome	5540
NC7: Coliphage NC7	NC_007834	Phage chromosome	5540
NC1: Coliphage NC1	NC_007832	Phage chromosome	5540
NC11: Coliphage NC11	NC_007835	Phage chromosome	5540
ID22: Coliphage ID22	NC_007829	Phage chromosome	5540
S13: Enterobacteria phage S13	NC_001424	Phage chromosome	5540
phiX174: Coliphage phiX174	NC_001422	Phage chromosome	5540
WA11: Coliphage WA11	NC_007843	Phage chromosome	5541
WA4: Coliphage WA4	NC_007841	Phage chromosome	5540
ID34: Coliphage ID34	NC_007830	Phage chromosome	5540
NC41: Coliphage NC41	NC_007838	Phage chromosome	5540
NC56: Coliphage NC56	NC_007840	Phage chromosome	5540
WA10: Coliphage WA10	NC_007842	Phage chromosome	5540
NC51: Coliphage NC51	NC_007839	Phage chromosome	5540
ID45: Coliphage ID45	NC_007831	Phage chromosome	5540
*SARS coronavirus* TW1	AY283796	Virus chromosome	30137
*SARS coronavirus* Sin2679	AY283797	Virus chromosome	30132
*SARS coronavirus* Sin2748	AY283798	Virus chromosome	30137
*SARS coronavirus* Sin2774	AY283794	Virus chromosome	30137
*SARS coronavirus* Sin2500	AY291451	Virus chromosome	30155
*SARS coronavirus* Urbani	AY278741	Virus chromosome	30153
*SARS coronavirus* Sin2677	AY283795	Virus chromosome	30131
*SARS coronavirus* BJ01	AY278488	Virus chromosome	30151
*SARS coronavirus* HKU-39849	AY278491	Virus chromosome	30168
*SARS coronavirus* CUHK-W1	AY278554	Virus chromosome	30162
*SARS coronavirus*	NC_004718	Virus chromosome	30178
*SARS coronavirus* CUHK-Su10	AY282752	Virus chromosome	30162
*Murine hepatitis virus* strain 2	AF201929	Virus chromosome	31724
*Murine hepatitis virus* strain Penn 97-1	AF208066	Virus chromosome	31558
*Murine hepatitis virus* strain ML-10	AF208067	Virus chromosome	31681
*Murine hepatitis virus* strain A59	NC_001846	Virus chromosome	31806
*Porcine epidemic diarrhea virus*	NC_003436	Virus chromosome	28435
*Avian infectious bronchitis virus*	NC_001451	Virus chromosome	28004
*Feline infectious peritonitis virus*	NC_002306	Virus chromosome	29776
*Human coronavirus* 229E	NC_002645	Virus chromosome	27709
*Bovine coronavirus* strain Quebec	AF220295	Virus chromosome	31546
*Bovine coronavirus* strain Mebus	u00735	Virus chromosome	31477
*Bovine coronavirus* isolate BCoV-LUN	AF391542	Virus chromosome	31473
*Bovine coronavirus*	NC_003045	Virus chromosome	31473

Taken together, such amazing landscapes (Figure 6, 7) can only be revealed by using the unique UGFA method, under the notions of “universal genome fingerprint map (UGFM)” of “total genetic component configuration (TGCC)” based “systematic comparative genomics (SCG)”. Namely, these data are more than enough to prove the concepts and tools (UGFM, UGFM-TGCC, and UGFM-TGCC-SCG) (Figure 5) effective and powerful in handling such real-world diverse genomes in-one-sitting. Most importantly, the representatives are elegantly plotted as beautiful and meaningful UGFM-TGCC-SCG visions (Figure 6, 7), explicitly demonstrating the scope and power of the unique comprehensive methods developed in the present study. Remarkably, we re-emphasize that the combined concept and tool of “UGFM-TGCC-SCG”, namely the “universal genome fingerprint map (UGFM)” of “total genetic component configuration (TGCC)” based “systematic comparative genomics (SCG)”, is distinguished from any other traditional methods of comparative genomics. This is simply because all genomes of interest crossing diverse genetic components (chromosomes, plasmids, and phages, if applicable) and diverse biological categories (bacteria, archaeal bacteria, viruses) are much less or even no homology at all (Figure 6, 7), which should be incredibly challenging to any conventional methods based on the traditional homology analysis. In fact, all documented researches so far about comparative genomics were automatically based on the assumption that there should be at least one reference for those very close relatives in question; otherwise, they would not bother to do comparison. However, in our case, we focus exactly on the opposites: much less or even no homology at all. We have demonstrated the successful usage of the UGFM-TGCC-SCG tool (Figure 6, 7) in comparing such diverse genetic components and diverse biological categories, regardless of the format of objects and the extent of divergences. Clearly, this is one of the core concepts and the most priority aim in the present study.

### 6. Quantitative Analysis of the Outcome Dataset of Genome Fingerprint Analysis

The difference between two genomes of interest, whose genome fingerprints are distinguished by one of the visions of UGFM, UGFM-TGCC, and UGFM-TGCC-SCG, can be further quantitatively discussed as follows.

#### 6.1. The geometric center and geometric mean of the genome fingerprint map

First, we define the geometric center (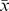,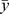,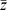) as a unique digital indicator for its genome fingerprint map. Accordingly, the geometric center (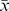,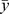,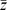) and the standard deviation of all coordinates (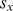,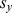,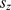) can be calculated (**5**) by GenomeFingerprinter.exe from a given genome sequence (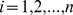) (the length of an entire genome sequence is usually greater than hundreds of base pairs).
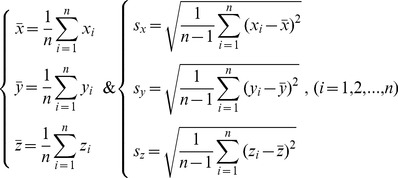
(5)


Second, we define the geometric mean (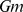) (**6**) of the geometric center of a given genome fingerprint map.
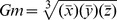
(6)


Note that the definition of 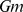 has two-fold meanings: one is algebraically calculating the geometric-mean value of the three means (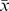,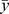,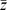), the other is geometrically defining the side-length value of a cube that is roughly equivalent to the cuboid volume, which is created by the values of geometric center starting from and rotating around the origin in the three-dimensional space. Accordingly, the values (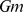,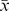,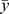,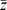) are not the absolute ones but carry the symbols (minus or plus), corresponding to the geometric center of the genome fingerprint map in the same three-dimensional space, namely within the scope of geometrical analysis.

#### 6.2. The Euclidean distance and differentiate rate between two genomes

To directly compare two genomes of interest, we define (**7**) the Euclidean distance (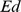), the differentiate rate (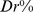), and the weighted differentiate rate (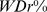) between two genomes in pairs, which are calculated based on the geometric means of the geometric centers of genome fingerprint maps. Again, the values (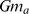,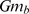,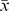,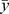,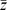) are not the absolute ones but carry the symbols (minus or plus) corresponding to their geometric centers of genome fingerprint maps in the same three-dimensional space.
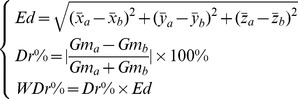
(7)


#### 6.3. Examples of the quantitative comparison between two genomes

As examples, thirty chromosomes (Table 1) give twenty-nine pairs of comparison (Table 3) as the representatives for illustrating the principles. As such, the rules can be summarized from these examples (Table 3). In general, the differentiate rates (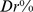) vary from family to family; and the values of 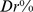 start from least at strain/species level (<50%) to higher at genus level (<500%) to even higher at beyond family level (<1500%). Of course, there are numerous outliers under certain situations (Table 3) with challenging values in terms of either the differentiate rate (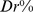), or the weighted differentiate rate (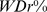), or the Euclidean distance (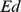).

**Table 3:** The quantitative analysis of representative taxa used in this study.

Taxon^a^	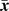	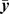	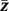	*Gm*	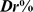 b	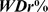 b	*Ed* b
*Escherichia coli* SMS-3-5	−723.50	3286.50	−25173.50	3911.76	3.36	38846.52	11544.34
*Escherichia coli* K-12/MG1655	−686.50	1944.50	−36639.51	3657.07	6.39	3171.28	496.33
*Escherichia coli* K-12/DH10B	−626.50	1452.50	−36613.51	3217.80	10.25	35994.85	3512.41
*Escherichia coli* K-12/W3110	254.00	−1905.00	−37151.01	2619.59	36.42	304004.04	8348.03
*Escherichia coli* UTI89	−3518.50	1648.50	−30606.51	5620.22	321.11	2987021.60	9302.08
*Escherichia coli* BL21 (DE3) pLysSAG	−299.00	−2237.00	−38421.01	−2951.00	42.76	535251.30	12518.87
*Escherichia coli* O55:H7/CB9615	4072.00	3474.00	−28174.01	−7359.17	22.74	97703.92	4296.20
*Escherichia coli* CFT073	1205.00	3302.00	−24979.00	−4632.12	202.03	88805749.64	439557.42
*Methanococcus voltae* A3	−6408.50	−970.50	414491.71	13711.64	25.10	587771.06	23419.60
*Methanosphaera stadtmanae* 3091	4145.50	7328.50	395302.72	22900.28	1467.93	16494288.43	11236.41
*Sulfolobus islandicus* Y.G.57.14	5251.00	−3846.00	394896.15	−19979.20	19.39	451075.63	23268.54
*Sulfolobus islandicus* Y.N.15.51	−7837.50	757.50	413575.65	−13490.82	25.28	667551.43	26407.22
*Sulfolobus islandicus* M.14.25	644.00	−2081.00	388729.15	−8046.40	6.42	5292.47	824.63
*Sulfolobus islandicus* M.16.4	476.50	−1916.50	387938.65	−7075.85	16.03	12422689.29	775145.76
*Haloarcula marismortui* 43049	401.00	865.00	−387202.12	−5121.13	30.68	11239486.19	366386.62
*Haloarcula marismortui* 43049-II	−1343.00	−717.00	−20823.07	−2716.74	216.56	73553959.06	339653.28
*Halobacterium salinarum* R1	−874.00	1275.00	−360470.18	7378.43	1.24	432.16	347.72
*Halobacterium sp*.NRC-1	−851.50	1213.50	−360811.68	7197.28	19.19	936045.35	48776.09
*Halogeometricum boringquense* 11551	−2079.00	1844.00	−312055.11	10615.71	1.58	275711.29	174157.24
*Halomicrobium mukohataei* 12286	1900.50	−1177.50	−486140.66	10284.83	42.76	2879398.22	67330.93
*Halomonas elongate* 2581	−6598.00	4630.00	−552680.14	25654.04	221.76	137405406.45	619606.27
*Haloquadratum walsbyi* 16790	−2613.00	5233.00	66913.02	−9708.09	36.36	17055884.77	469043.29
*Halorhabdus utahensis* 12940	5125.50	4368.50	−402065.63	−20802.81	160.79	16003171.72	99528.02
*Halorhodospira halophilia* SL1	−26901.01	54859.02	−481632.18	89243.66	133.41	8899042.85	66706.24
*Halorubrum lacusprofundii* 49239	−2129.50	−2139.50	−457398.67	−12773.06	42.39	17797581.80	419838.30
*Halorubrum lacusprofundii* 49239-II	−1716.50	−2140.50	−37560.57	−5167.70	54.44	31479912.34	578208.73
*Haloterrigena turkmenica* 5511	−3902.00	−2238.00	−615765.16	−17519.47	215.81	200314105.28	928180.28
*Halothermothrix orenii* H 168	255.00	3329.00	312389.12	6424.66	291.91	126733403.82	434156.90
*Halothiobacillus neapolitanus* c2	2821.00	6576.00	−121748.05	−13120.26	17.10	6022573.34	352284.18
*Natrinema pellirubrum* 15624	−4316.50	−3111.50	−473826.67	−18531.35	/	/	/

a The taxa with GenBank_ID are cross-listed in Table 1.

b The Euclidean distance (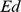),differentiate rate (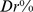), and weighted differentiate rate (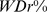) are calculated according to the formula (**7**) by using two adjacent sequences in pairs; and the resultant is listed at the same upper row as the first sequence of the pairs, as shown by the last two rows.

For example, the two chromosomes of two strains (*Sulfolobus islandicus* M.14.25 and M.16.4) with subtle variations in their genome fingerprint maps (Figure 2, A) can be quantitatively differentiated through the distinct values of geometric center (644.00, −2081.00, 388729.15) *vs.* (476.50, −1916.50, 387938.65) and geometric mean (−8046.40) *vs*. (−7075.85), clearly indicating they are not identical. The differentiate rate between them is only 6.42% (Table 3). Evidently, such two strains have distinct values of geometric center and geometric mean of the genome fingerprint maps, but the differentiate rate is less than 10%. Indeed, they had been characterized as two distinct but close strains within the same species, *Sulfolobus islandicus*. In addition, there are four close strains in this species, with differentiate rates ranging between 6.42% and 25.28% (Table 3).

Another example compares two very distant strains (beyond family level), *Sulfolobus islandicus* Y.G.57.14 *vs. Methanosphaera stadtmanae* DSM 3091 (Figure 2, C) with the following diverse data: geometric center (5251.00, −3846.00, 394896.15) *vs*. (4145.50, 7328.50, 395302.72), geometric mean (−19979.20) *vs.* (22900.28). Moreover, the differentiate rate between them is 1467.93% (Table 3), which is much greater than those values at genus level. These data together confirm that the two strains are farther divergent beyond the family level.

Furthermore, there are three remarkable exceptions (Table 3). First, within the same one strain, there are two chromosomes; and the differentiate rate between the two chromosomes is at least close to the values between two species or genera, implying that such two chromosomes are divergent and each independently impacts on the same strain. For instance, the differentiate rates of *Halorubrum lacusprofundii* 49239 *vs*. 49239-II (42.39%) and *Haloarcula marismortui* 43049 *vs*. 43049-II (30.68%), respectively, are close to certain values of the differentiate rates (e.g., 42.76%, 36.36%, 54.44%) at genus level within the same family *Halobacteriaceae*. Second, within the same species, *Escherichia coli*, three strains (BL21(DE3), CB9615, CFT073) are extraordinary because the differentiate rate between UT189 and BL21(DE3) is 321.11%, which is extremely out of the ranges (3.36%∼36.42%) defined by the ordinary members in the same species; and it is even much greater than the value of 25.10% between two external genera (*Methanococcus voltae* A3 and *Methanosphaera stadtmanae* 3091) in other family. Third, within the same family, *Halobacteriaceae*, the differentiate rates among different genera vary between 17.10% and 291.91%. Putting together, these data probably indicate that such strains (particularly containing more than one chromosome) have been continuously growing and absorbing new composites so that they are potentially developing into a new species. Moreover, from family to family, the genus levels are not within the same range of divergence in terms of the differentiate rates, implying no possibility of setting up a universal boundary for simply distinguishing all taxa.

Most importantly, although the differentiate rate (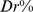) is concise and efficient for most cases (Table 3), we also note that the weighted differentiate rate (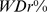) is more accurate to deal with outliers, giving more reasonable inference through the cross-validations after having factored the differentiate rate (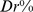) with the Euclidean distance (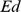). For example, two genera (*Halogeometricum boringquense* 11551 *vs*. *Halomicrobium mukohataei* 12286) seem very similar due to the tiny differentiate rate (1.58%) by chance resulting from the very similar values of 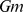 (10615.71 *vs*. 10284.83), but they are actually quite different in terms of their geometric centers (−2079.00, 1844.00, −312055.11) *vs*. (1900.50, −1177.50, −486140.66), resulting in larger values of the weighted differentiate rate (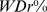 = 275711.29) and the Euclidean distance (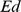 = 174157.24), which are essentially close to the extents that distinguished the divergences between other genera in the same family. Thus, we suggest that either 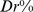 or 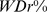 can be generally referred to an inference (the first is concise while the latter is accurate); but for outliers arisen, both of them have to be cross-referenced explicitly.

## Discussion

We believe that performing what we called the systematic comparative genomics based on the geometrical analysis of genome sequences, instead of the pair-wisely base-to-base comparison, is a priority task in the post-genomic era. To our knowledge, however, no attention as what we did in the present study has been paid to compare a number of genomes crossing genetic components (chromosomes, plasmids, and phages) and biological categories (bacteria, archaeal bacteria, and viruses) with far divergence over large size range. In particular, no method for creating the unambiguous genome fingerprint (GF) has been documented; neither the universal genome fingerprint analysis (UGFA), nor the total genetic component configuration (TGCC), nor the systematic comparative genomics (SCG) has been proposed; nonetheless, no method for quantitatively differentiating genome sequences has been developed based on using the outcome dataset of genome fingerprint analysis.

Remarkably, the genome sequences crossing diverse genetic components (chromosomes, plasmids, and phages) or crossing diverse biological categories (bacteria, archaeal bacteria, and viruses) have much less or even no homology, which should be incredibly challenging to any conventional methods that are principally based on the pair-wisely base-to-base homology analysis. In other words, no conventional method can compare such diverse genetic components and biological categories in-one-sitting, as what we did in the present study. Therefore, it would be impossible to compare other conventional methods with our comprehensive methods as a whole system: the method of genome fingerprinting (*GenomeFingerprinter*), the method of universal genome fingerprint analysis (UGFA) (including the UGFM, UGFM-TGCC, and UGFM-TGCC-SCG tools), and the method of quantitative analysis (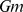,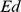,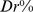,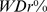) for the outcome dataset of the genome fingerprint analysis. In the present study, however, we have tried our best to compare partial features between our methods and others that are partly related to ours, as well as briefly discuss the future perspectives of quantitative analysis for using the outcome dataset of the universal genome fingerprint analysis.

### 1. *GenomeFingerprinter vs*. Zplotter

#### 1.1. Validity

The Zplotter program [16] is not used for the creation of what we called “genome fingerprint (GF)”. In fact, although some coordinates from the Zplotter program were used to produce hundreds of graphs (as open rough Z-curves) of microbial genomes that were documented as a database [17], there were no stable unique features in terms of the so-called genome fingerprints. For example, when we re-plotted the visions of *Halobacterium* sp. NRC-1 genome sequence (NC_002607) using the Zplotter’s coordinates of either z_n_' or z_n_, respectively, to present an open rough Z-curve (data not shown), those visions themselves were quite different from one another due to the wavelet transform in the algorithm of Zplotter program [16]. In contrast, our method presented a unique circular vision with accurate and delicate genome fingerprints for the same sequence (data not shown). Again, note that using the z_n_, coordinates gave a similar vision to ours, except that it was in an open rough Z-curve with less features; while using the z_n_' coordinates created a completely different vision from ours (data not shown). We conclude that our *GenomeFingerprinter* method provides more accurate and delicate coordinates than the Zplotter program does, and therefore is valid for the subsequent applications that have been established by the Z-curve analysis. Of course, one should beware of choosing whether z_n_ from our method or z_n_' from the Zplotter program when referring to specific questions.

#### 1.2. Reliability

We found a major problem when using the Zplotter program to handle circular genome sequences with cutting-point errors. In fact, for example, the same circular sequence of *Halobacterium* sp. NRC-1 (NC_002607) but with two different cutting-points (e.g., NC_002607_RC was re-cut at 700 kbps) were incorrectly presented as different visions by using the Zplotter’s coordinates; whereas both scenarios were exactly shown as the same vision by using our method (data not shown). The reasons for such differences come from that the Zplotter program was designed for a linear sequence [16] and its algorithm depends on counting the absolute numbers of bases starting from the “first” base in a given linear sequence. Meanwhile, when a sequence was deposited as a linear form (regardless of the original linear or circular form), the documented first base was usually not guaranteed to be the real first one. Taken together, the same circular sequence with cutting-point error changing its real “first” base can result in a quite different vision by using the Zplotter program. In contrast, our method was initially created for a circular sequence (Figure 1) but has been proved also valid for a linear one as exemplified earlier. This is not only because the linear form is a specific form of circular one, but also because the formula (**1**) described earlier ensures that our method measures the relative distance in a circular form, rather than the absolute numbers of bases counting from the “first” base in a linear sequence. In other words, our method has been proved valid for both circular and linear forms regardless of where the cutting-point is (i.e., where the “first” base is), overriding any possible cutting-point errors.

#### 1.3. Adaptability

We further emphasize the scientific foundations for the reason why it is critical to deal with circular genomes, which has been overlooked in literatures.

Theoretically, most microbial genomes are in circular strands, which protect them from natural degradation due to relatively simple structures. In other words, the circular form is much more stable than its linear form in living cells. In most cases, the circular genomes and their linear forms usually change into one another when and only when they are at certain functioning stages of living cells, such as the rolling-model replication and the plasmid-mediated conjunction. Most importantly, the circular and linear forms are both genetically and physiologically functioning in a coordinated way for a given genome in a given living microbe. That is, their forms are interchangeable when responding to real living conditions. Therefore, we can catch up the circular status of genomes during their life cycles.

Technically, different groups world-wide have not been unified yet to guarantee that all genome sequences are deposited in their correct forms. In fact, most sequences deposited in public databases (such as GenBank) so far are neither in their natural orders of starting from the real “first” base, nor in the assumed direction from 5′ to 3′. We thus have to tackle such cutting-point errors, as illustrated by those examples earlier. Fortunately, the RD formula (**1**) in our method can virtually treat an arbitrary linear sequence as a circular one (Figure 1), avoiding impacts of any possible cutting-point errors exist in the public deposited sequences.

Informatively, the closed (circular form) genome fingerprints carry much more sensitive information, considering genome-wide comparative genomics at the genome fingerprint level (Figure 3). Our method can precisely calculate a set of three-dimensional coordinates for a given circular or linear sequence with or without correct cutting-point, which accordingly can present a stable unique genome fingerprint map and further guarantee the validity of the universal genome fingerprint analysis.

To conclude, the *GenomeFingerprinter* method has great advantages over the Zplotter program in creating unambiguous sets of coordinates, which is valid to the subsequent applications that have been established by the Z-curve analysis [18], [19], [20], [21], [22], [23].

### 2. *Genome Fingerprinter vs*. Mauve

#### 2.1. Efficiency

The Mauve program (a typical algebraic-type approach), combining both computing and plotting, is commonly used for pair-wisely base-to-base comparison and visualization [14], [15]. However, it has difficulty when dealing with a number of larger genome sequences due to its inner computational constraints, either too slow or memory overflow. In contrast, our method can rapidly calculate and visualize, separately, tens of large genomes, and is much faster than the Mauve program in terms of the time complexity [*O*(*n*) *vs*. *O*(*n^2^*) ] (data not shown). Furthermore, with our method under our hardware conditions (physical 2 Gb memory and 32-bits graphic software), more than one hundred genome sequences can be elegantly plotted in-one-sitting (Figure 7). Only plotting numerous larger graphics in-one-sitting would cause memory overflow. Most importantly, our method performs calculation and visualization separately, which not only ensures higher performance efficiency for a large set of genomes, but also provides output dataset for the universal genome fingerprint analysis (Figure 2, 3, 4, 5, 6, 7) and quantitative analysis (Table 3).

#### 2.2. Prediction

The Mauve program [14], [15] can only visualize what a sequence is, but cannot predict what it should be without one reference sequence or specific pre-knowledge. In contrast, our method provides the universal genome fingerprint map (UGFM) (either the P-GFM or the S-GFMs), which can intuitively identify the unique genome features such as the genome-wide evolution events and the replication *ori* points (Figure 3) that have been characterized in literatures [22], [23], [24], [25].

#### 2.3. Compatibility

The universal genome fingerprint analysis (UGFA) predicted the subtle variations (Figure 3, C, D, G) indicating the genome-wide evolution events (Figure 3, C). We then used the Mauve program to pair-wisely compare two chromosomes and confirmed such events (data not shown), demonstrating that the UGFA method could rapidly predict the evolution events while the Mauve program could precisely confirm such predictions. Thus, we recommend that the UGFA method and the Mauve program be compatible partners, taking advantages of ours for rapid intuitive prediction in general (Figure 3, 6) and of Mauve’s for slow precise confirmation in detail, particularly focusing on the targeted fragments’ gain, lose, and rearrangement (data not shown).

Likewise, among nineteen genomes (Figure 6), including six chromosomes and thirteen plasmids with large size range (6 Kbp∼4 Mbp) belonging to the four strains crossing four genera of halophilic Archaea (Table 1), the rare homology was mapped only by the progressiveMauve mode [14] (data not shown); whereas the Mauve mode [15] failed in such a comparison because it stopped due to no essential homology, as we predicted beforehand. Yet, the Mauve mode [15] worked well with the subset of either thirteen plasmids or six chromosomes, respectively, confirming their partial homology (data not shown). In other words, the UGFM-TGCC-SCG tool can not only handle the exceptional situations for a large set of genomes, but also facilitate the effective integration of the Mauve program into performing the so-called systematic comparative genomics among a large set of genome sequences crossing diverse genetic components (chromosomes, plasmids, and phages) and diverse biological categories (bacteria, archaeal bacteria, and viruses) with far divergence (less or no homology) over large size range (e.g., 6 Kbp∼4 Mbp) (Figure 6, 7). Meanwhile, the progressiveMauve mode [14] can be compatible to the UGFA method (including the UGFM, UGFM-TGCC, and UGFM-TGCC-SCG tools), whereas the Mauve mode [15] cannot, but still can be used to partially deal with the subsets of genomes in question.

Taken together, we conclude that the UGFA method (including the UGFM, UGFM-TGCC, and UGFM-TGCC-SCG tools) has advantages over the Mauve program [14], [15] in dealing with a set of genomes of less or no homology. Particularly, we recommend that any components with farther divergence be rapidly pre-screened out by using the UGFM-TGCC-SCG tool, which could guide the selection of subsets in question for the subsequent comparisons by using the appropriate mode of Mauve program [14], [15].

### 3. The Quantitative Analysis of the Outcome Dataset of Genome Fingerprint Analysis

Obviously, the main purpose of the present study is to develop a novel method, *GenomeFingerprinter*, taking a geometric approach to intuitively visualize a genome sequence in order to distinguish numerous genome sequences through their intuitive images. Namely, it is designed to extract the meaningful information but reduce the massive noise from the original millions of base pairs of genome sequences. Accordingly, there is no intention to go backward to perform extensive statistic analysis on such massive discrete data in a traditional way. Rather, we have developed the method of quantitative analysis by using the outcome dataset of genome fingerprint analysis. In particular, we have defined the geometric center (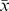,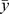,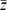) and its following geometric mean (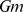) of a given genome fingerprint map to determine the Euclidean distance (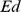), the differentiate rate (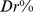), and the weighted differentiate rate (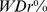) in order to quantitatively describe the difference between two genomes of comparison. In fact, the applications with certain examples (Table 3) have demonstrated that the differentiate rates generally vary from family to family starting from least at strain/species level (<50%) to higher at genus level (<500%) to even higher at beyond family level (<1500%), which seem promising to be as the basic rules for setting up the general boundaries at certain levels of taxonomical units.

However, we would remind its limitation at current status. As stated earlier, those data (Table 3) demonstrated that, from family to family, the genus levels were not within the same range of divergence in terms of the differentiate rates, implying no possibility of setting up a universal boundary for simply distinguishing all taxa. We thus recommend that the inference based on the (weighted) differentiate rate and the Euclidean distance be conducted under clear biological contexts because of two major reasons. First, such inferences should not be made solely based on the differentiate rates when dealing with outliers encountered (Table 3). For instance, *Halogeometricum boringquense* 11551 (NC_014729) *vs*. *Halomicrobium mukohataei* 12286 (NC_013202), two genera seemed very similar (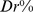 = 1.58%) by chance resulting from the very similar values of 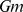, but they were actually quite different in terms of their geometric centers, which were also verified by the large values of the Euclidean distance and the weighted differentiate rate (Table 3). Second, it is still unclear to determine a precise boundary corresponding to the taxonomical hierarchy because we found that the differentiate rates of outliers dramatically varied (Table 3), implying no such a boundary could be possibly determined under current knowledge. We thus remind that there is a huge gap to be fulfilled before eventually setting up the upper and lower boundaries in the real-world for different levels of taxa (strains, species, genera, families, and beyond).

Meanwhile, we have only established the method of quantitative analysis to simply compare two genomes in pairs (Table 3). To make intensive statistic analysis about a number of genomes as one sample or two samples, we suggest that a sophisticated method be developed first, which is beyond the scope of the present study. For example, considering very fewer genome sequences available within certain taxonomic units resulting in very small sizes of samples, the traditional empirical methods of statistical inference and hypothesis testing (such as the normal *z*-test and student’s *t*-test) would not be appropriate. As such, we suggest that the permutation-based randomization test, such as bootstrap, should be developed for such statistic analyses in order to better use the outcome dataset of the genome fingerprint analysis. To this end, for example, the geometric center, the Euclidean distance and the (weighted) differentiate rate as potential statistical estimators should be kept worthy of being further explored with more real-world data at large scale in future.

### Conclusions

We have developed the methodology of what we called the systematic comparative genomics based on the genome fingerprint and the universal genome fingerprint analysis. First, we have created a method, *GenomeFingerprinter*, to unambiguously produce the three-dimensional coordinates from a sequence, followed by one three-dimensional plot and six two-dimensional trajectory projections, to illustrate the genome fingerprint of a given genome sequence. Second, we have developed a set of concepts and tools (3D-P, 2D-TP, GF, GFM, P-GFM, S-GFM, UGFM, TGCC, UGFM-TGCC, SCG, UGFM-TGCC-SCG), and thereby established a method called the universal genome fingerprint analysis (UGFA). Particularly, we have demonstrated that the UGFM, UGFM-TGCC, and UGFM-TGCC-SCG tools have great advantages over other conventional methods. Third, we have constructed a method of quantitative analysis to compare two genomes by using the outcome dataset of genome fingerprint analysis. Specifically, we have defined the geometric center (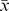,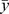,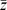) and its following geometric mean (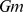) for a given genome fingerprint map, followed by the Euclidean distance (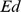), the differentiate rate (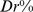) and the weighted differentiate rate (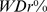) to quantitatively describe the difference between two genomes of comparison. Moreover, we have demonstrated the applications through case studies on various genome sequences crossing diverse genetic components (chromosomes, plasmids, and phages) and crossing diverse biological categories (bacteria, archaeal bacteria, and viruses) with far divergence (less or no homology) over large size range (4 kilo-∼5 mega-base pairs per sequence), giving tremendous insights into the critical issues in microbial genomics and taxonomy. We therefore anticipate that these comprehensive methods can be widely applied to the so-called systematic comparative genomics at large scale in the post-genomic era.

### Materials

Genome sequences used in this study were downloaded from NCBI or derived from this study, which are listed in Table 1 and Table 2.

### Methods

We have implemented our method into an in-house script (GenomeFingerprinter.exe). It will be available upon request to the corresponding author. The programs of Zplotter (v1.0) and Mauve (v2.3.1) used in this study can be downloaded from links: Zplotter.exe at http://tubic.tju.edu.cn/zcurve/and Mauve at http://gel.ahabs.wisc.edu/mauve/. To plot graphics from coordinates, any graphic tool can be used.

## Supporting Information

Table S1 **Features of genome sequences from bacteria and archaeal bacteria.**
(DOC)

Table S2 **Features of genome sequences from phages and viruses.**
(DOC)
