# Supplementary material for: GenomeFingerprinter: The Genome Fingerprint and the Universal Genome Fingerprint Analysis for Systematic Comparative Genomics
Source: PLoS One. 2013 Oct 29;8(10):e77912. doi: 10.1371/journal.pone.0077912 (PMC3812135; doi:10.1371/journal.pone.0077912)
Supplement: Table S1 — Features of genome sequences from bacteria and archaeal bacteria. (DOC) [file pone.0077912.s001.doc]

**Table S1. Features of genome sequences from bacteria and archaeal bacteria**

| **Species and Strain** | **Sequence ID** | **Type** | **Size (bps)** |
| --- | --- | --- | --- |
| **Downloaded from FTP.ncbi.nlm.nih.gov [GenBank]** | | | |
| *Escherichia coli* K-12/W3110 | AC_000091  NC_007779 | Chromosome | 4646332 |
| *Escherichia coli* K-12/DH10B | NC_010473 | Chromosome | 4686137 |
| *Escherichia coli* K-12/MG1655 | NC_000913 | Chromosome | 4639675 |
| *Escherichia coli* BL21 (DE3) pLysSAG | NC_012947 | Chromosome | 4570938 |
| *Escherichia coli* O55:H7/CB9615 | NC_013941 | Chromosome | 5386352 |
| *Escherichia coli* UTI89 | NC_007946 | Chromosome | 5065741 |
| *Escherichia coli* CFT073 | NC_004431 | Chromosome | 5231428 |
| *Escherichia coli* SMS-3-5 | NC_010498 | Chromosome | 5068389 |
| *Sulfolobus islandicus* M.14.25 | NC_012588 | Chromosome | 2608832 |
| *Sulfolobus islandicus* M.16.4 | NC_012726 | Chromosome | 2586647 |
| *Sulfolobus islandicus* Y.N.15.51 | NC_012623 | Chromosome | 2812165 |
| *Sulfolobus islandicus* Y.G.57.14 | NC_012622 | Chromosome | 2702058 |
| *Methanococcus voltae* A3 | NC_014222 | Chromosome | 1936387 |
| *Methanosphaera stadtmanae* DSM 3091 | NC_007681 | Chromosome | 1767403 |
| *Halomonas elongate* DSM 2581 | NC_014532 | Chromosome | 4119315 |
| *Halorhodospira halophilia* SL1 | NC_008789 | Chromosome | 2716716 |
| *Halorhabdus utahensis* DSM 12940 | NC_013158 | Chromosome | 3161321 |
| *Halothermothrix orenii* H 168 | NC_011899 | Chromosome | 2614977 |
| *Halothiobacillus neapolitanus* c2 | NC_013422 | Chromosome | 2619785 |
| *Halogeometricum boringquense* DSM 11551 | NC_014729 | Chromosome | 2860838 |
| *Haloterrigena turkmenica* DSM 5511 | NC_013743 | Chromosome | 3944596 |
| *Natrinema pellirubrum* DSM 15624 | NC_019962 | Chromosome | 3844629 |
| *Haloquadratum walsbyi* DSM 16790 | NC_008212 | Chromosome | 3177244 |
| *Halorubrum lacusprofundii* ATCC49239 | NC_012029 | Chromosome | 2774371 |
| *Halorubrum lacusprofundii* ATCC49239 | NC_012028 | Chromosome | 533457 |
| *Haloarcula marismortui* ATCC43049 | NC_006396 | Chromosome | 3176463 |
| *Haloarcula marismortui* ATCC43049 | NC_006397 | Chromosome | 292165 |
| *Haloarcula marismortui* ATCC43049 | NC_006389 | plasmid pNG100 | 33779 |
| *Haloarcula marismortui* ATCC43049 | NC_006390 | plasmid pNG200 | 33930 |
| *Haloarcula marismortui* ATCC43049 | NC_006391 | plasmid pNG300 | 40086 |
| *Haloarcula marismortui* ATCC43049 | NC_006392 | plasmid pNG400 | 50776 |
| *Haloarcula marismortui* ATCC43049 | NC_006393 | plasmid pNG500 | 134574 |
| *Haloarcula marismortui* ATCC43049 | NC_006394 | plasmid pNG600 | 157519 |
| *Haloarcula marismortui* ATCC43049 | NC_006395 | plasmid pNG700 | 416420 |
| *Halomicrobium mukohataei* DSM 12286 | NC_013202 | Chromosome | 3154923 |
| *Halomicrobium mukohataei* DSM 12286 | NC_013201 | plasmid pHmuk01 | 225032 |
| *Haloferax vocanii* DS2 | NC_013967 | Chromosome | 2888440 |
| *Haloferax vocanii* DS2 | NC_013964 | plasmid pHV3 | 444162 |
| *Haloferax vocanii* DS2 | NC_013965 | plasmid pHV2 | 6450 |
| *Haloferax vocanii* DS2 | NC_013966 | plasmid pHV4 | 644869 |
| *Haloferax vocanii* DS2 | NC_013968 | plasmid pHV1 | 86308 |
| *Halobacterium* sp.NRC-1 | NC_002607 | Chromosome | 2014239 |
| *Halobacterium* *salinarum* R1 | NC_010364 | Chromosome | 2000962 |
| **Derivatives created in this study [based on those sequences from GenBank]** | | | |
| *Escherichia coli* K-12/W3110-91.1.1 | 91.1.1 | Chromosome fragment | 227694 |
| *Escherichia coli* K-12/W3110-91.1.61 | 91.1.61 | Chromosome fragment | 324260 |
| *Escherichia coli* K-12/W3110-91.6.59 | 91.6.59 | Chromosome fragment | 410186 |
| *Escherichia coli* K-12/W3110-91.F7 | 91.7 | Chromosome fragment | 953958 |
| *Escherichia coli* K-12/MG1655-913.1.77 | 913.1.77 | Chromosome fragment | 331163 |
| *Escherichia coli* K-12/MG1655-913.5.57 | 913.5.57 | Chromosome fragment | 408963 |
| *Escherichia coli* CFT073-4431.1.70 | 4431.1.70 | Chromosome fragment | 401260 |
| *Escherichia coli* UTI89-7946.4.7 | 7946.4.7 | Chromosome fragment | 518065 |
| *Escherichia coli* K-12/DH10B -10473.1.74 | 10473.1.74 | Chromosome fragment | 325622 |
| *Escherichia coli* K-12/DH10B -10473.4.57 | 10473.4.57 | Chromosome fragment | 412818 |
| *Escherichia coli* SMS-3-5-10498.4.86 | 10498.4.86 | Chromosome fragment | 331536 |
| *Escherichia coli* BL21 (DE3) pLysSAG-12947.F1 | 12947.1 | Chromosome fragment | 1759795 |
| *Escherichia coli* BL21 (DE3) pLysSAG-12947.1.50 | 12947.1.50 | Chromosome fragment | 470050 |
| *Escherichia coli* BL21 (DE3) pLysSAG-12947.F5 | 12947.5 | Chromosome fragment | 43254 |
| *Escherichia coli* O55:H7/CB9615-13941.F1 | 13941.1 | Chromosome fragment | 1915479 |
| *Escherichia coli* O55:H7/CB9615-13941.2.60 | 13941.2.60 | Chromosome fragment | 267039 |
