# Supplementary material for: GenomeFingerprinter: The Genome Fingerprint and the Universal Genome Fingerprint Analysis for Systematic Comparative Genomics
Source: PLoS One. 2013 Oct 29;8(10):e77912. doi: 10.1371/journal.pone.0077912 (PMC3812135; doi:10.1371/journal.pone.0077912)
Supplement: Table S2 — Features of genome sequences from phages and viruses. (DOC) [file pone.0077912.s002.doc]

**Table S2**. Features of genome sequences from phages and viruses

| **Species and Strain** | **Sequence ID** | **Type** | **Size (bps)** |
| --- | --- | --- | --- |
| **Downloaded from FTP.ncbi.nlm.nih.gov [GenBank]** | | | |
| WA5: Coliphage WA5 | NC_007847 | Phage chromosome | 5737 |
| ID11: Coliphage ID11 | NC_006954 | Phage chromosome | 5737 |
| WA3: Coliphage WA3 | NC_007845 | Phage chromosome | 5700 |
| WA2: Coliphage WA2 | NC_007844 | Phage chromosome | 5700 |
| ID41: Coliphage ID41 | NC_007851 | Phage chromosome | 5737 |
| NC10: Coliphage NC10 | NC_007854 | Phage chromosome | 5687 |
| WA6: Coliphage WA6 | NC_007852 | Phage chromosome | 5687 |
| ID12: Coliphage ID12 | NC_007853 | Phage chromosome | 5687 |
| NC13: Coliphage NC13 | NC_007849 | Phage chromosome | 5737 |
| NC2: Coliphage NC2 | NC_007848 | Phage chromosome | 5737 |
| NC6: Coliphage NC6 | NC_007855 | Phage chromosome | 5687 |
| ID52: Coliphage ID52 | NC_007825 | Phage chromosome | 5698 |
| ID8: Coliphage ID8 | NC_007846 | Phage chromosome | 5700 |
| G4: Enterobacteria phage G4 | NC_001420 | Phage chromosome | 5737 |
| ID2: Coliphage ID2 | NC_007817 | Phage chromosome | 5644 |
| WA14: Coliphage WA14 | NC_007857 | Phage chromosome | 5644 |
| ID18: Coliphage ID18 | NC_007856 | Phage chromosome | 5644 |
| WA45: Coliphage WA45 | NC_007822 | Phage chromosome | 6242 |
| ID21: Coliphage ID21 | NC_007818 | Phage chromosome | 6242 |
| NC28: Coliphage NC28 | NC_007823 | Phage chromosome | 6239 |
| ID62: Coliphage ID62 | NC_007824 | Phage chromosome | 6225 |
| NC35: Coliphage NC35 | NC_007820 | Phage chromosome | 6213 |
| NC29: Coliphage NC29 | NC_007827 | Phage chromosome | 6439 |
| NC3: Coliphage NC3 | NC_007826 | Phage chromosome | 6273 |
| alpha3: Enterobacteria phage alpha3 | DQ085810 | Phage chromosome | 6177 |
| WA13: Coliphage WA13 | NC_007821 | Phage chromosome | 6242 |
| phiK: Coliphage phiK | NC_001730 | Phage chromosome | 6263 |
| ID32: Coliphage ID32 | NC_007819 | Phage chromosome | 6245 |
| NC19: Coliphage NC19 | NC_007850 | Phage chromosome | 5737 |
| NC16: Coliphage NC16 | NC_007836 | Phage chromosome | 5540 |
| NC5: Coliphage NC5 | NC_007833 | Phage chromosome | 5540 |
| NC37: Coliphage NC37 | NC_007837 | Phage chromosome | 5540 |
| ID1: Coliphage ID1 | NC_007828 | Phage chromosome | 5540 |
| NC7: Coliphage NC7 | NC_007834 | Phage chromosome | 5540 |
| NC1: Coliphage NC1 | NC_007832 | Phage chromosome | 5540 |
| NC11: Coliphage NC11 | NC_007835 | Phage chromosome | 5540 |
| ID22: Coliphage ID22 | NC_007829 | Phage chromosome | 5540 |
| S13: Enterobacteria phage S13 | NC_001424 | Phage chromosome | 5540 |
| phiX174: Coliphage phiX174 | NC_001422 | Phage chromosome | 5540 |
| WA11: Coliphage WA11 | NC_007843 | Phage chromosome | 5541 |
| WA4: Coliphage WA4 | NC_007841 | Phage chromosome | 5540 |
| ID34: Coliphage ID34 | NC_007830 | Phage chromosome | 5540 |
| NC41: Coliphage NC41 | NC_007838 | Phage chromosome | 5540 |
| NC56: Coliphage NC56 | NC_007840 | Phage chromosome | 5540 |
| WA10: Coliphage WA10 | NC_007842 | Phage chromosome | 5540 |
| NC51: Coliphage NC51 | NC_007839 | Phage chromosome | 5540 |
| ID45: Coliphage ID45 | NC_007831 | Phage chromosome | 5540 |
| *SARS coronavirus* TW1 | AY283796 | Virus chromosome | 30137 |
| *SARS coronavirus* Sin2679 | AY283797 | Virus chromosome | 30132 |
| *SARS coronavirus* Sin2748 | AY283798 | Virus chromosome | 30137 |
| *SARS coronavirus* Sin2774 | AY283794 | Virus chromosome | 30137 |
| *SARS coronavirus* Sin2500 | AY291451 | Virus chromosome | 30155 |
| *SARS coronavirus* Urbani | AY278741 | Virus chromosome | 30153 |
| *SARS coronavirus* Sin2677 | AY283795 | Virus chromosome | 30131 |
| *SARS coronavirus* BJ01 | AY278488 | Virus chromosome | 30151 |
| *SARS coronavirus* HKU-39849 | AY278491 | Virus chromosome | 30168 |
| *SARS coronavirus* CUHK-W1 | AY278554 | Virus chromosome | 30162 |
| *SARS coronavirus* | NC_004718 | Virus chromosome | 30178 |
| *SARS coronavirus* CUHK-Su10 | AY282752 | Virus chromosome | 30162 |
| *Murine hepatitis virus* strain 2 | AF201929 | Virus chromosome | 31724 |
| *Murine hepatitis virus* strain Penn 97-1 | AF208066 | Virus chromosome | 31558 |
| *Murine hepatitis virus* strain ML-10 | AF208067 | Virus chromosome | 31681 |
| *Murine hepatitis virus* strain A59 | NC_001846 | Virus chromosome | 31806 |
| *Porcine epidemic diarrhea virus* | NC_003436 | Virus chromosome | 28435 |
| *Avian infectious bronchitis virus* | NC_001451 | Virus chromosome | 28004 |
| *Feline infectious peritonitis virus* | NC_002306 | Virus chromosome | 29776 |
| *Human coronavirus* 229E | NC_002645 | Virus chromosome | 27709 |
| *Bovine coronavirus* strain Quebec | AF220295 | Virus chromosome | 31546 |
| *Bovine coronavirus* strain Mebus | u00735 | Virus chromosome | 31477 |
| *Bovine coronavirus* isolate BCoV-LUN | AF391542 | Virus chromosome | 31473 |
| *Bovine coronavirus* | NC_003045 | Virus chromosome | 31473 |
